# Hatha yoga for acute, chronic and/or treatment-resistant mood and anxiety disorders: A systematic review and meta-analysis

**DOI:** 10.1371/journal.pone.0204925

**Published:** 2018-10-01

**Authors:** Nina K. Vollbehr, Agna A. Bartels-Velthuis, Maaike H. Nauta, Stynke Castelein, Laura A. Steenhuis, H. J. Rogier Hoenders, Brian D. Ostafin

**Affiliations:** 1 Lentis Psychiatric Institute, Center for Integrative Psychiatry, Groningen, the Netherlands; 2 University of Groningen, Faculty of Behavioral and Social Sciences, Department of Clinical Psychology and Experimental Psychopathology, Groningen, the Netherlands; 3 University of Groningen, University Medical Center Groningen, University Center for Psychiatry, Rob Giel Research Center, Groningen, the Netherlands; 4 Lentis Psychiatric Institute, Lentis Research, Groningen, the Netherlands; Brown University, UNITED STATES

## Abstract

**Background:**

The aim of this study was to systematically investigate the effectiveness of hatha yoga in treating acute, chronic and/or treatment-resistant mood and anxiety disorders.

**Methods:**

Medline, Cochrane Library, Current Controlled Trials, Clinical Trials.gov, NHR Centre for Reviews and Dissemination, PsycINFO and CINAHL were searched through June 2018. Randomized controlled trials with patients with mood and anxiety disorders were included. Main outcomes were continuous measures of severity of mood and anxiety symptoms. Cohen’s *d* was calculated as a measure of effect size. Meta-analyses using a random effects model was applied to estimate direct comparisons between yoga and control conditions for depression and anxiety outcomes. Publication bias was visually inspected using funnel plots.

**Results:**

Eighteen studies were found, fourteen in acute patients and four in chronic patients. Most studies were of low quality. For depression outcomes, hatha yoga did not show a significant effect when compared to treatment as usual, an overall effect size of Cohen’s *d* -0.64 (95% *CI* = -1.41, 0.13) or to all active control groups, Cohen’s *d* -0.13 (95% *CI* = -0.49, 0.22). A sub-analysis showed that yoga had a significant effect on the reduction of depression compared to psychoeducation control groups, Cohen’s *d* -0.52 (95% *CI* = -0.96, -0.08) but not to other active control groups, Cohen’s *d* 0.28 (95% *CI* = -0.07, 0.63) For studies using a follow-up of six months or more, hatha yoga had no effect on the reduction of depression compared to active control groups, Cohen’s *d* -0.14 (95% *CI* = -0.60, 0.33). Regarding anxiety, hatha yoga had no significant effect when compared to active control groups, Cohen’s *d* -0.09 (95% *CI* = -0.47, 0.30). The I^2^ and Q-statistic revealed heterogeneity amongst comparisons. Qualitative analyses suggest some promise of hatha yoga for chronic populations.

**Conclusions:**

The ability to draw firm conclusions is limited by the notable heterogeneity and low quality of most of the included studies. With this caveat in mind, the results of the current meta-analysis suggest that hatha yoga does not have effects on acute, chronic and/or treatment-resistant mood and anxiety disorders compared to treatment as usual or active control groups. However, when compared to psychoeducation, hatha yoga showed more reductions in depression. It is clear that more high-quality studies are needed to advance the field.

## Introduction

Worldwide, mood and anxiety disorders represent the two most common forms of mental disorders [1]. For example, it has been estimated that nearly 28% of Europeans and 55% of Americans experience one of these disorders during their life [2, 3]. Further, research has consistently shown a relation between these disorders, as demonstrated by their high rates of comorbid symptoms [4, 5]. Given that mood and anxiety disorders are characterized by the presence of negative affect [6], a number of theorists propose that difficulty in regulating negative affect represents a psychological mechanism for both disorders [7, 8]. Examples of difficulties in regulating negative affect include repetitive negative thinking [9–11] and emotional and behavioral avoidance [12].

A large proportion of individuals have *chronic forms* (over two years [13]) of mood and anxiety disorders. Chronicity is exhibited by approximately 25% of individuals with a mood disorder and 40% of individuals with an anxiety disorder [14]. Chronicity in these disorders is associated with higher health care use, lower social-economic functioning, reduction in work productivity, and lower quality of life compared to acute forms of disorders [15–18].

Cognitive-behavioral therapy and pharmacotherapy have been shown to improve acute symptoms [19–21], but the benefits of current first-line treatments for acute patients are modest, as indicated by medium to small effect sizes [19, 21] and rates of nonresponse to treatment, ranging from 19–34% [22, 23]. Chronically depressed patients experience even fewer benefits from therapy, as indicated by small effect size outcomes [24]. Epidemiological studies have suggested that anxiety disorders tend to be chronic at even higher rates than mood disorders [18, 25, 26]. A proportion of individuals with chronic forms of mood and anxiety disorders are those who are treatment-resistant. Although the field lacks a consensus of how to define treatment-resistance, there is some general agreement that treatment resistance involves no or only partial improvement from pre- to post-intervention (after treatment of adequate dose and duration [27–30]). It is estimated that 30–40% of patients recover with standard treatment and another 30–40% can be considered partially improved [29, 30]. In sum, the research reviewed above makes it clear that a large number of patients with mood and anxiety disorders do not (fully) respond to treatment, leaving many patients with chronic forms of these disorders. Therefore, it is important to continue searching for new approaches to target the difficulty of regulating negative affect and prevent chronicity in mood and anxiety disorders.

One new approach for treating mood and anxiety disorders is hatha yoga, a form of yoga that uses physical postures in combination with breathing and/or meditation practices [31]. Hatha yoga may be well suited as an intervention for mood and anxiety disorders given initial findings that hatha yoga helps to lessen psychological distress [32, 33]. Hatha yoga has also been shown to influence transdiagnostic processes underlying mood and anxiety disorders, such as repetitive negative thinking [34, 35] and avoidance of negative emotions [36–38]. Further, hatha yoga involves elements of physical exercise and meditation, both of which have been shown to be useful in treating psychological distress [39–41] and in influencing the transdiagnostic processes of repetitive negative thinking [42, 43] and avoidance of negative emotions [44, 45].

Yoga also holds promise as an intervention due to its acceptability. Intervention acceptability is an important variable given that the majority of individuals suffering from mood and anxiety disorders do not seek treatment [46] and that among treatment seekers, a substantial percent drop out of treatment [47, 48]. Evidence for the acceptability of yoga includes that it is widely embraced by the public [49] and is increasingly sought as a means of treating depression and anxiety [50].

The effects of yoga on mood and anxiety disorders have been reviewed by several authors [51–59]. These systematic reviews and two meta-analyses concluded that there is some evidence of yoga being effective for mood and anxiety disorders, but all mention serious methodological drawbacks of the included randomized controlled trials (RCTs). An important limitation of these reviews is that they included studies with both nonclinical and clinical samples, making it difficult to draw conclusions regarding the efficacy of yoga for patients. Additionally, neither the meta-analyses nor the systematic reviews examined the effects of yoga for chronic and/or treatment-resistant populations. Understanding the efficacy of yoga for these populations is important, given that these forms of mood and anxiety disorders are associated with poorer treatment outcomes and greater economic and other societal costs [24]. Another limitation of these reviews is that they included a diverse range of yoga interventions, ranging from meditation-only to complex interventions involving yoga postures, meditation, breath work, and lifestyle modifications. Such heterogeneity obscures the ability to draw conclusions about the benefits of yoga as it is difficult to distinguish which of the diverse forms are helpful and which are not.

We therefore conducted a systematic review and meta-analysis to answer two questions: (1) is hatha yoga an effective treatment for acute mood and anxiety disorders, and (2) is hatha yoga an effective treatment for chronic and/or treatment-resistant mood and anxiety disorders?

## Methods

This systematic review and meta-analysis was planned and conducted following the guidelines of the PRISMA statement [60].

### Search

A clinical librarian searched the existing literature for articles describing RCTs for yoga interventions in adult clinical samples with mood and anxiety disorders with the following search terms: (MH "Yoga" OR yog* OR asana* OR pranayama OR dhyana) (the last three being Sanskrit terms for the different components of yoga: postures, breathing exercises and meditation) AND (MH "Depression" OR MH "Depressive Disorder+" OR MH "Anxiety+" OR MH "Anxiety Disorders+" OR MH "Mood Disorders+" OR depress* OR dysthym* OR anx* OR MDD OR GAD OR mood OR affective). We searched both relevant Medical Subject Headings and free-text terms. The following databases were searched until June 2018: Medline, Cochrane Library, Current Controlled Trials, Clinical Trials.gov, NHR Centre for Reviews and Dissemination, PsycINFO and CINAHL. Additionally, reference lists of relevant review papers extracted from the database search were manually reviewed.

### Selection of trials

Two independent reviewers (AABV and NKV) selected studies if they: (1) were an RCT, comparing a yoga intervention to a wait-list control group, treatment as usual (TAU) or an active control (e.g., exercise or relaxation), (2) included a hatha yoga intervention that incorporated physical postures based on yoga theory, possibly also including meditative practices and/or breathing practices, (3) included a majority of patients with major depression, dysthymic disorder, generalized anxiety disorder, social anxiety disorder, or panic disorder (as these three anxiety disorders are considered closely related according to DSM-V [13]), diagnosed with the criteria of the International Classification of Disease 10 (ICD-10 [61]) or the Diagnostic and Statistical Manual, Fourth Edition (DSM-IV [62]), or older versions of the DSM (including DSM-I [63], DSM-II [64] and DSM-III [65], using older terms referring to the three included anxiety disorders, including anxiety neurosis and psychoneurosis [66] or older terms referring to various types of depression, including neurotic and reactive depression [66]), (4) included adult samples (ages 18–65 years), (5) were written in the English language, (6) did not describe the same study (or part of a study) of another article, and (7) included either a continuous measure of improvement or a dichotomous measure of remission of mood and/or anxiety symptoms at both pre- and post-intervention, using validated self-report scales (e.g., Beck Depression Inventory, BDI [67]), or clinician-rated scales (e.g., Hamilton Rating Scale for Depression, HRSD [68]).

Mindfulness-based stress reduction (MBSR) and mindfulness-based cognitive therapy (MBCT) interventions were excluded because these have more focus on sitting meditation and body scans, with yoga being only a small part of these interventions. No restrictions were made regarding yoga tradition, intervention length or frequency. Studies that paired yoga with other interventions such as treatment as usual were also included.

### Data extraction

Major characteristics of the included studies were coded by two independent reviewers (AABV and NKV), including outcome variables (symptoms of depression and/or anxiety), patient characteristics (diagnosis, based on which diagnostic interview, inpatients or outpatients), intervention characteristics (type of yoga, length of the sessions, duration of the intervention, homework), control group characteristics (type of control group, length of the session, duration of the intervention, homework), and general characteristics (co-interventions, number of patients per group). The population of the study was coded as acute or chronic and/or treatment-resistant. We defined *chronic* as: having a mood or anxiety disorder for over two years, without full remission [13]. We defined *treatment-resistant* as: having received at least two standard interventions of adequate dose and duration (pharmacotherapy or a psychological intervention), without full remission [30]. If the majority of a study sample was diagnosed with a chronic and/or treatment-resistant depression or anxiety disorder, we classified this study as chronic and/or treatment-resistant. Disagreements were discussed with a third reviewer (HJRH) until agreement was reached.

### Effect size calculations

The primary outcome measure in the meta-analysis was the standardized mean change in symptoms of depression and/or anxiety. For primary outcome measures, the standardized mean change (Cohen’s *d*) between the baseline and post-treatment assessment for each treatment condition (intervention and control groups) in the studies was calculated. The following equation was used [69]:

$$Cohen’sd=(M_{T1}–M_{T0})/SD_{T0}$$

with *M*_T1_ as the mean of the post-treatment outcome measure and *M*_T0_ the mean of the baseline outcome measure. *SD*_T0_ indicates the standard deviation of the baseline outcome measure. For the follow-up effect sizes, *M*_T1_ represented the mean of the follow-up outcome measure and *M*_T0_ the mean of the post-treatment outcome measure, whilst *SD*_T0_ stood for the standard deviation of the post-treatment outcome measure.

The overall effect size comparing the yoga intervention group to the control group, was calculated with the subsequent equation:

$$\text{Cohen}’\text{s}\;d\;(treatment\;vs.\;control)=d_{\text{intervention}}–d_{\text{control}}$$

with *d*_intervention_ as the effect size of the intervention group and *d*_control_ for the effect size of the control group.

The Standard Error (S.E.) of the Cohen’s *d* was calculated, used to weigh effect sizes when combining studies, so that large studies are considered more important than small studies in the analysis, using the following equation [70]:

$$\text{S}.\text{E}.\;\text{of}\;\text{Cohen}’\text{s}\;d=\frac{2\left[1-r\right]}{n}+\frac{d^{2}}{2\left(n-1\right)}$$

with *n* representing the number of participants in the treatment arm, *r* representing for the baseline to post-treatment correlation and *d* representing the effect size as calculated by Cohen’s *d*. This was calculated separately for the yoga group and the control group.

To calculate the S.E. for the overall Cohen’s *d* for yoga vs. control, the S.E. of the control group must be added to the S.E. of the yoga group, and subsequently the square root of this value must be computed.

When a study used more than one relevant active intervention as a control condition, we used a combined effect size of the two active interventions because two separate outcomes from the same study (and the same participants) cannot be entered into one meta-analytic pooled effect size, as the outcomes are not independent from each other and the errors are correlated [71]. The combined effect size for these studies was calculated with the following equation [71]:

$$\text{Combined effect size}\;(yoga\;vs.\;active\;control^{1}\;and^{}\;yoga\;vs.\;active\;control^{2})=\frac{1}{2}\left[d1+d2\right]$$

with *d*_1_ representing the effect size of the yoga group compared to the first active intervention and *d*_2_ representing the effect size for the yoga group compared to the second active intervention.

To calculate a combined standard error, the following equation was used [71]:

$$\text{Combined standard error}=S.E._{1}+S.E._{2}+2r\surd S.E._{1}\surd S.E._{2}$$

with S.E._1_ representing the Standard Error of the effect size of the yoga group compared to the first active intervention and S.E._2_ representing the Standard Error of the effect size of the yoga group compared to the second active intervention. The *r* represents the correlation between the two active control conditions.

### Quality assessment

To assess the quality of the studies, two independent raters (AABV and NKV) assessed the studies using the Clinical Trials Assessment Measure for psychological treatments (CTAM [72]), a rating scale designed to assess the quality of psychological interventions in mental health care. The scale assesses six aspects of the design of the trial: (1) sample size and how the sample was recruited, (2) allocation of treatment, (3) assessment of the outcome measures, (4) type of control group, (5) statistical analyses, and (6) description of the intervention. The CTAM score ranges from 0 to 100, with a score above 65 considered to be adequate. Disagreements were discussed with a third reviewer (HJRH) until agreement was reached. The scale had a good blind inter-rater agreement of .96 and a sufficient internal consistency with a Cronbach’s alpha of .70 [73].

### Statistical analysis

Statistical analyses were performed using R software, with the metaphor package [74]. A meta-analysis using a random effects model was applied to estimate direct comparisons between yoga and control conditions for both depression and anxiety outcomes. Heterogeneity was assessed using the Q and I^2^ statistic [75], to examine and quantify whether the variability in estimates of effect sizes from similar studies excels the variation expected from sampling error. If the Q statistic is non-significant, this indicates that there is no significant heterogeneity and Cohen’s *d* can be reliably interpreted. I^2^ was interpreted and quantified as low, moderate, and high to values of 25%, 50%, and 75% [76]. Publication bias was assessed using the Egger’s test and funnel plots [77]. If the p-value of the Egger’s test is 0.1 or lower, and the funnel plots appear asymmetric, there is an indication for publication bias.

## Results

### Search results

**Fig 1:**
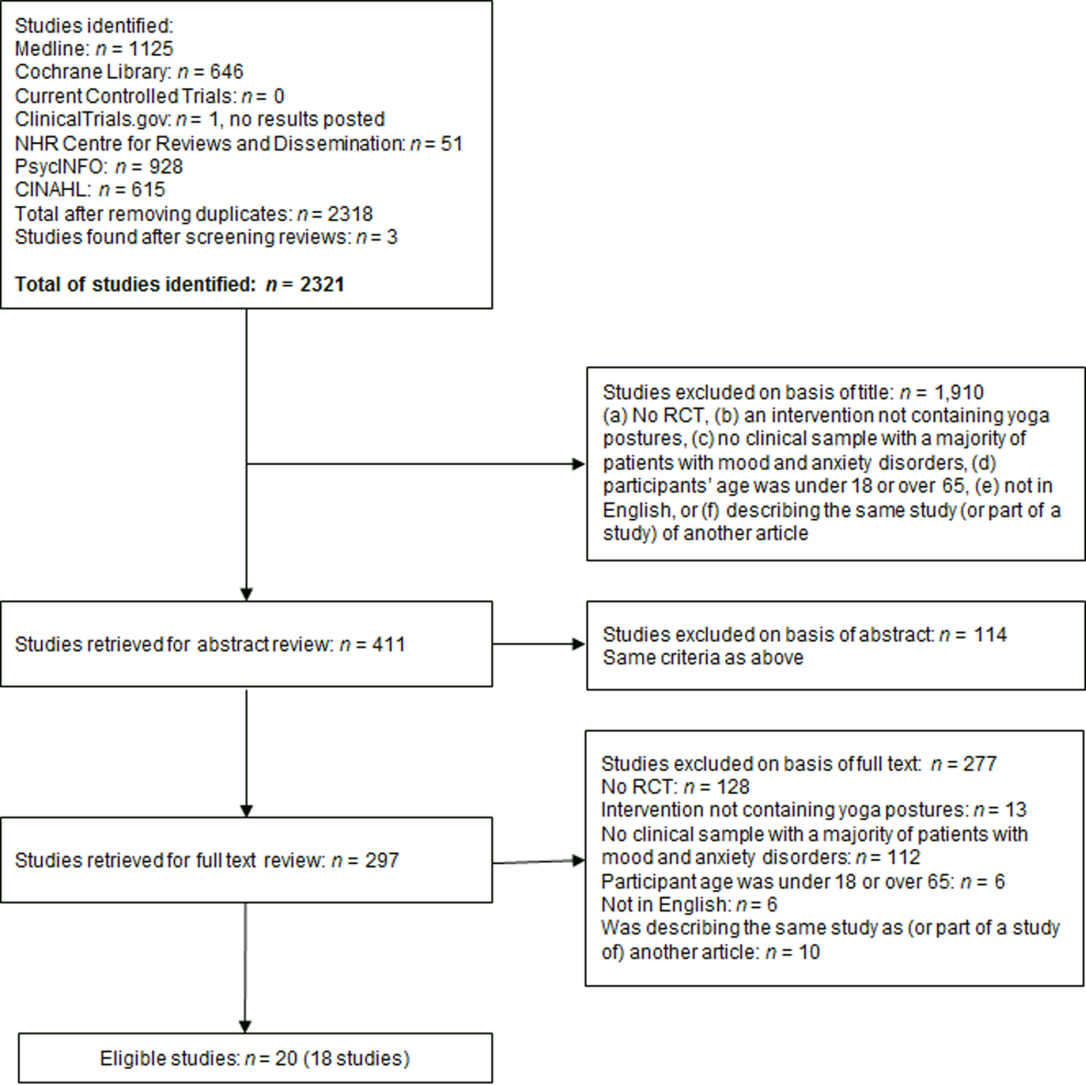
Flow chart of the study selection process and included studies.

An overview of the selection process and included studies is given in Fig 1. The search in the aforementioned databases resulted in 2,318 articles for screening, with three additional articles identified via cross-reference searching. Of the 2,321 articles, 409 abstracts were screened. A further 113 articles were excluded based on their abstract, using the criteria mentioned in the Methods section. After a full-text screening of the remaining articles, 18 studies (in 20 articles) were included.

### Characteristics of included studies

The final 18 trials were divided into studies with acute clinical populations (*n* = 14) and studies with chronic and/or treatment-resistant clinical populations (*n* = 4). Selected characteristics of the included studies are presented in Table 1 for acute populations and in Table 2 for chronic populations. The total number of participants was 1,532 with sample sizes ranging from 12 to 620 participants (mean = 85). Approximately 561 participants received a yoga intervention (one study did not report the number of participants per group [78]). The majority of the participants was female (78.7%, *n* = 1,072), with seven studies including only women [34, 35, 78–82]. Three studies did not report the gender of the participants [83–85]. The mean age was 36.6 years (SD 9.1; range 22.1–50.4). The studies were performed in the United States [35, 79–82, 86–90], India [78, 83–85, 91, 92], Sweden [93], and Germany [94].

**Table 1:** Selected characteristics of the included studies for acute populations.

Study	Total patients (yoga group), diagnosis	Diagnosis by	Co- interventions	Intervention groups, length, frequency, duration, amount of home practice		Length of intervention, follow-up	Outcome measures 1. Primary 2. Secondary	Results		CTAM
				Treatment	Control			Short term	Long term	
Broota & Dhir, 1990 [91]	30 (10*) adults with neurotic/ reactive depression (baseline scores unknown), outpatients from a psychiatry department	Psychiatrist	Antidepressant medication	Broota Relaxation Technique (BRT, postures, breathing), 1x 20 min/day, home practice not reported	1. PMR, 1x 20 min/ day 2. Control group, narrating about problems, 1x 20 min/ day	3 days	1. Symptoms of depression (Symptom check list)	1. Sig dif favoring BRT over control 2. BRT slightly superior to JPR		36
Falsafi, 2016 [88]	90 (30*) students with depression and/or anxiety disorder (baseline BDI 20.0/21.1/20.2; HAM-A 21.2/22.2/21.1), recruitment at the university	Health care professio-nal	Regular treatment (medication and/or psychotherapy)	Hatha Yoga (postures, meditation), 1x 75 min/week, daily 20 min home practice	1. Mindfulness training, 1x 75 min/week, daily 20 min home practice 2. Control group (no intervention)	8 weeks, 12 weeks	1. Symptoms of depression (BDI) 2. Symptoms of anxiety (HAM-A)	1. Sig dif favoring yoga and mindfulness over control, no sig dif between yoga and mindfulness 2. Sig dif favoring yoga and mindfulness over control, no sig dif between yoga and mindfulness	1. Sig dif favoring yoga and mindfulness over control, no sig dif between yoga and mindfulness 2. Sig dif favoring yoga and mindfulness over control, no sig dif between yoga and mindfulness	37
Field et al., 2013 [79]	92 (46*) prenatally depressed women (MDD, dysthymia) (baseline CES-D 33.0/35.1; STAI 53.4/55.0), community sample	SCID	None	Yoga (postures), 1x 20 min/ week, home practice with manual and dvd, not reported how frequent	Social support group, 1x 20 min/week	12 weeks, postpartum (1–3 weeks post birth)	1. Symptoms of depression (CES-D, EPDS) 2. Symptoms of anxiety (STAI)	1. No sig group dif 2. No sig group dif	1. No sig group dif 2. No sig group dif	67
Field et al., 2012 [80]	84 (unclear*) prenatally depressed women (MDD, dysthymia) (baseline CES-D 28.35/24.08/22.65; STAI 50.0/44.19/ 42.38), community sample	SCID	Mostly none (95%)	Yoga (postures), 1x 20 min/week, home practice not reported	1. Massage, 1x 20 min/week 2. Standard prenatal care	12 weeks	1. Symptoms of depression (CES-D) 2. Symptoms of anxiety (STAI)	1. Sig dif favoring yoga over control group, no sig dif between yoga and massage 2. Sig dif favoring yoga over control group, no sig dif between yoga and massage		64
Helgadót-tir et al., 2016 & 2018 [93, 97]	620 (106*) adults with a depressive disorder or anxiety disorder (baseline MADRS 21.5), community sample	MINI	Standard treatment for depression	Yoga (postures), 3x 55 min/week	1. Intermediate-level aerobics, 3x 55 min/week 2. More strenuous aerobics, 3x 55 min/week 3. Treatment as usual	12 weeks, 1 year	1. Symptoms of depression (MADRS)	1. Sig dif favoring yoga group over control, trend towards a sig diff favoring yoga over moderate exercise group, no sig diff between yoga and vigorous exercise group	1. Sig dif favoring yoga group over control, sig dif favoring yoga over moderate exercise, no sig dif between yoga and vigorous exercise group	68
Kinser et al., 2013 & 2014 [86, 34]	27 (15*) women with MDD or dysthymia (baseline PHQ-9 14.8/18.3; STAI 52.5/55.1), community sample	MINI	Usual depression care	Gentle Hatha yoga (postures, breathing, relaxation), 1x 75 min session/ [90](89)(89)week, daily home practice with dvd	Health education sessions, 1x 75 min/ week, weekly review handout at home	8 weeks, 1 year	1. Symptoms of depression (PHQ-9) 2. Symptoms of anxiety (STAI)	1. No sig group dif 2. No sig group dif	1. Sig dif favoring yoga over control 2. No sig group dif	40
Mitchell et al., 2012 [81]	24 (12*) prenatally depressed women (MDD) (baseline CES-D 22.42/27.5), community sample	SCID	Not reported	Yoga (postures), 2x 20 min/ week, home practice not reported	Parenting education, 2x 20 min/week	12 weeks	1. Symptoms of depression (CES-D)	1. Sig dif favoring yoga group over control		39
Parthasa-rathy et al., 2014 [78]	45 (unclear*) women with anxiety disorder (baseline TAS 114.4/114.46/ 114.27), from a tertiary care center	Unclear	Not reported	Yoga (postures, breathing, relaxation), 45 min/day	1. Integrated yoga module (more postures, breathing, relaxation), 45 min/day 2. Control group with no special activities	8 weeks	1. Symptoms of anxiety (TAS)	1. Reduction in both yoga groups, not control group. Sig dif favoring integrated yoga module over yoga		32
Prathi- kanti et al., 2017 [89]	38 (20*) adults with major depression (baseline BDI 22.4), community sample	MINI	1 participant took psychotherapy	Hatha yoga (postures, breathing, meditation), 2x 90 min / week, home practice not reported	Education modules on yoga history and philosophy, 16x 90 min / week, home practice not reported	8 weeks	1. Symptoms of depression (BDI)	1. Sig dif favoring yoga group over control		49
Sahasi et al., 1989 [84]	91 (38*) adults with anxiety neurosis (baseline scores unknown), outpatients from a psychiatric center	Psychiatrist	Not reported	Yoga (postures, breathing, meditation), 7x 40 min session/ week (5x with instructor, 2x at home)	Diazepam (no dose or frequency given)	3 months	1. Symptoms of anxiety (IPAT)	1. Sig reduction for yoga group, not for control group		34
Sarubin et al., 2014 [94]	60 (22*) adults with MDD (baseline HAM-D 22.04), unclear recruitment	SCID	Quetiapine or escitalopram	Hatha yoga (no description given), 1x 60 min / week, home practice not reported	Control group (no yoga)	5 weeks	1. Symptoms of depression (HAM-D)	1. No sig group dif		35
Schuver & Lewis, 2016 [35]	40 (20*) depressed women (baseline BDI 22.36), community sample	SCID	Usual depression care	Mindfulness-based yoga (postures, breathing, meditation), by a DVD, 2x 60–75 min / week	Walking, with a DVD, 2x 60 min / week	12 weeks, 1 month	1. Symptoms of depression (BDI)	1. No sig group dif	1. No sig group dif	37
Tolahu-nase et al., 2018 [92]	58 (29*) adults with MDD (baseline BDI 26.96/28.10), outpatients from a psychiatry department	Unclear	Routine drug treatment for at least 6 months	Yoga- and meditation-based lifestyle interventions (postures, breathing, meditation, lectures on lifestyle), 5x 120 min / week	Control group (no yoga)	12 weeks	1. Symptoms of depression (BDI)	1. Sig dif favoring yoga group over control		52
Uebela-cker et al., 2016 [82]	20 (12*) pregnant women with depression (major or minor depression) (baseline QIDS 11.17/11.5), community sample	SCID	1 person took antidepressant medication	Gentle yoga (postures, breathing, meditation), 1x 75 min / week, home practice encouraged	Mom-baby wellness workshop, 1x 75 min / week	9 weeks	1. Symptoms of depression (QIDS, EPDS)	1. No sig group dif		42

*Note*. BAI, Beck Anxiety Inventory; BDI, Beck Depression Inventory; CBT, Cognitive Behavioral Therapy; CES-D, Center for Epidemiological Studies Depression Scale; CIS, Clinical Interview Schedule; dif, difference; EPDS, Edinburgh Postnatal Depression Scale; fu, follow-up; GAD, Generalized Anxiety Disorder; HAM-A, Hamilton Rating Scale for Anxiety; HAM-D, Hamilton Rating Scale for Depression; IPAT, Institute for Personality and Ability Testing [anxiety scale]; LoE, Level of Evidence; MADRS, Montgomery-Asberg Depression Rating Scale; MDD, Major Depressive Disorder; min, minutes; MINI, MINI International Neuropsychiatric Interview; PHQ, Patient Health Questionnaire; PMR, Progressive Muscle Relaxation; QIDS, Quick Inventory of Depressive Symptomatology; SCID, Structured Clinical Interview for DSM-IV; sig, significant; STAI, State-Trait Anxiety Questionnaire; TAS, Taylor’s Anxiety Scale;

* Number of patients that received the yoga intervention.

**Table 2:** Selected characteristics of the included studies for chronic and/or treatment-resistant populations.

Study	Total patients [yoga group], diagnosis	Diagnosis by	Co- interventions	Intervention groups, length, frequency, duration, amount of home practice		Length of intervention, follow-up	Outcome measures 1. Primary 2. Secondary	Results		CTAM
				Treatment	Control			Short term	Long term	
Butler et al., 2008 [87]	52 (17*) adults with dysthymia, double depression, (chronic) MDD (baseline HAM-D 15.87/12.33/ 15.81), community sample	SCID	Medication, no psychotherapy	Meditation and Hatha yoga (postures, breathing, meditation), 1x 2hr/week, daily home practice encouraged with audiocassettes (30 min/day, 6x /week)	1. Group therapy with hypnosis, 1x 1.5 hr/week 2. Control group, psychoeducation	12 weeks, 9 months	1. Symptoms of depression (HAM-D; CDRS-SR) 2. MDE; Remission > 2 months	1. No sig group dif	1. No sig group dif 2. MDE: sig dif favoring both groups over control; Remission: sig dif favoring yoga over control	60
Gupta et al., 2013 [83]	12 (6*) adults with GAD (baseline HAM-A 30.83/32.0), outpatients from institute for yoga and naturopathy	Unclear	Not reported	Yoga (postures, breathing), 1 hr/ day, home practice not reported	Naturopathy, 2x 30 min/day	3 weeks	1. Symptoms of anxiety (HAM-A)	1. No sig group dif, more improvement in yoga group		29
Uebela-cker et al., 2017 [90]	122 (63*) adults with major depressive disorder (baseline QIDS 12.87), community sample (2/3 chronic)	SCID	Antidepressant medication (95–100%), psychotherapy (40%)	Hatha yoga (postures, breathing, meditation), at least 1 x 80 min / week, home practice encouraged	Healthy living workshop, 1–2 x 60 min / week	10 weeks, 3 and 6 months	1. Symptoms of depression (QIDS, PHQ-9)	1. No sig group dif	3 months: sig dif favoring yoga group over control 6 months: sig dif favoring yoga group over control	82
Vahia et al., 1973, stage 2 [85]	27 (15*) adults with psychoneur-osis, absence of response to conventional treatments (baseline TAS 25.53/29.83), outpatients from a hospital	Unclear	Placebo tablet, support, reassurance	Yoga (postures, breathing, meditation), 1 hr, 5 days / week	Relaxation resembling yoga, 1 hr, 5 days / week	4 weeks (at least)	1. Symptoms of anxiety (TAS)	1. Sig more improvement in yoga than control		56

*Note 1*. CBT, Cognitive Behavioral Therapy; CDRS-SR, Cornell Dysthymia Rating Scale; dif, difference; HAM-A, Hamilton Rating Scale for Anxiety; HAM-D, Hamilton Rating Scale for Depression; LoE, Level of Evidence; MDE, Major Depressive Episode; min, minutes; MINI, MINI International Neuropsychiatric Interview; QIDS, Quick Inventory of Depressive Symptomatology; SCID, Structured Clinical Interview for DSM-IV; sig, significant; TOP, Treatment Outcome Package; sign, significant; TAS, Taylor’s Anxiety Scale;

* Number of patients that received the yoga intervention.

Ten studies were aimed at patients with an acute mood disorder: major depressive disorder [35, 81, 82, 89, 92, 94], major depressive disorder and dysthymia [79, 80, 86] and neurotic or reactive depression [91]. Two studies were aimed at patients with an acute anxiety disorder: anxiety neurosis or psychoneurosis [84] and an anxiety disorder that was not further specified [78]. Two studies included patients with a depressive disorder and/or an anxiety disorder [88, 93].

We found one study in patients with a chronic major depressive disorder (dysthymia, double depression, and major depressive disorder; defined as having experienced symptoms for over two years, without significant remission of two months or more [87]), one study in which almost two thirds of the sample had chronic major depression (defined as reporting symptoms over the past two years with absence of remission over two months [90]), one study in patients with treatment-resistant psychoneurosis or depression (defined as absence of response to conventional treatments, without defining these treatments [85]) and one study in which the majority of patients (58.33%) had a chronic generalized anxiety disorder (defined as 3–5 years of symptoms [83]). In one study the sample was described as chronically depressed using the definition of being depressed at least from the onset of pregnancy [80]. As this did not meet our definition of chronicity, this study was considered as aimed at acute patients.

Diagnoses were made with the Structured Clinical Interview for the DSM-IV [95; 35, 79–81, 82, 87, 90, 94] and the MINI International Neuropsychiatric Interview [96; 86, 89, 93]. In three studies, the diagnosis was made by a psychiatrist or other health care professional, based on the DSM-IV or an earlier version, without mentioning how this diagnosis was established [84, 88, 91]. In four studies, it was unclear how and by whom the DSM-diagnosis was established [78, 83, 85, 92].

Large differences were observed between the trials in terms of treatment dosage, amount of homework practice, comparison groups, and follow-up duration. Length of the intervention varied between three days [91] and twelve weeks [35, 79–81, 84, 87, 92, 93]. The amount of practice ranged from 120 minutes a day [92] to 20 minutes a week [79, 80]. In ten studies, daily (home) practice was encouraged, at least for five days a week [78, 82, 84–88, 90–92]. In five studies, it was unclear how often or whether participants were encouraged to practice at home [79–81, 89, 94]. Most studies did not include a longer follow-up period, except for seven studies that used a follow-up postpartum [79], at 4 weeks [35, 88], at 3 months [90], at 6 months [90], at 9 months [87], and after one year [34, 97].

Most studies reported baseline and post-treatment means but five studies only reported the mean change of the outcome measure [83, 84], the percentage of change [90, 91], the difference between time points [97] or means at follow-up only [86, 34]. These authors were contacted for the separate baseline and post-treatment means in order to be able to homogeneously calculate the effect sizes for all studies. Two studies did not report the standard deviations of the means [78, 89] and one study did not report the means and standard deviations for the post-treatment assessment [94]. These authors were contacted for the means and standard deviations in order to be able to calculate the effect sizes for all studies. Three authors (or colleagues in their department) were unable to provide the requested data [83, 84, 91], and one author did not respond despite repeated efforts to make contact [78]. The data from these four studies were excluded from the meta-analysis.

Only one study used dichotomous outcomes of depression [87]. If studies reported multiple assessment points after baseline, only those assessed immediately after treatment were used. The baseline to post-treatment correlation and the correlation between active control conditions [93] was not reported in the included articles. In line with recommendations from previous research [39], we assumed a conservative baseline to post-treatment correlation of 0.7 for all studies. For the correlation between active control conditions, we assumed a correlation of 0.5 in line with recommendations by Borenstein [71]. A sensitivity analysis around this correlation provided support for its use.

### Outcome measures

Fifteen RCTs assessed symptoms of depression, using the Beck Depression Inventory [35, 88, 89, 92], Center of Epidemiological Studies Depression Scale [79–81], Quick Inventory of Depressive Symptomatology [82, 90], Edinburgh Postnatal Depression Scale [79, 82], Hamilton Depression Rating Scale [87, 94], Patient Health Questionnaire [86, 90], Cornell Dysthymia Rating Scale-Self Report [87], Montgomery-Asberg Depression Rating Scale [93], Profile of Mood States [79], Symptom Sign Inventory [84], or a personalized assessment of symptoms [91]. Eight RCTs assessed symptoms of anxiety, using the State-Trait Anxiety Inventory [79, 80, 86], the Hamilton Anxiety Rating Scale [83, 88], the Taylor’s Anxiety Scale [78, 85], and the IPAT Anxiety Scale [84]. Only one RCT assessed remission rates, using the Structured Clinical Interview for the DSM-IV to obtain remission rates [87]. Since for this study we also had continuous outcomes available we used the highest ranked continuous outcome measure for our analyses.

When a study assessed the outcome variable using multiple measurement scales [79, 82, 87, 90], we selected the most suitable depression scale, based on a preferred hierarchy of depression scales, determined post-hoc (Table 3). This hierarchy was based on the available evidence on the reliability and validity of the scale as an assessment tool for depression, and was determined in consensus with NKV and AABV. For Butler and colleagues [87], the preferred scale was the Hamilton Depression Rating Scale, for Field and colleagues [79], this was the Center of Epidemiological Studies Depression Scale, and for both studies of Uebelacker and colleagues [82, 90], this was the Quick Inventory of Depressive Symptomatology.

**Table 3:** Hierarchy of preferred depression measurement scales.

Ranking	Measurement Scale	Source for ranking
1	Hamilton Depression Rating Scale	[98]
2	Quick Inventory of Depressive Symptomatology	[98]
3	Patient Health Questionnaire-9	[99]
4	Center of Epidemiological Studies Depression Scale	[100]
5	Profile of Mood States	[101]
6	Edinburgh Postnatal Depression Scale	[102]
7	Cornell Dysthymia Rating Scale	[103]

### Comparison groups

For the meta-analysis, we divided the comparison groups into active interventions and treatment as usual (TAU) interventions. One study used ‘no intervention’ as control group [88]. Given that at least a quarter of participants received TAU, we considered the control group of this study as TAU. We compared yoga with a general grouping of *active interventions* and, when there were enough studies to do so, with sub-groups of active interventions, consisting of *psychoeducation control groups* (e.g., interventions controlling only for non-specific factors, for instance healthy living classes) and *other active interventions* (e.g., interventions controlling for non-specific factors as well as therapeutic factors, for instance mindfulness or walking).

Four studies included in the meta-analysis used more than one comparison group which could not be entered together into one pooled effect size [71]. In two studies [80, 88], the first comparison group was an active intervention and the second comparison group was TAU, so these groups are included in separate analyses (yoga vs. active control and yoga vs. TAU). One study [93] used a TAU condition, as well as two active interventions of different intensity (intermediate-level aerobics and more strenuous aerobics). As both active interventions were similar with a different intensity, we used a combined effect size for this comparison [71]. One study [87] used two active comparison groups. One group was a psychoeducation group and the other group also consisted of other therapeutic ingredients (group therapy with hypnosis) so these groups were included in the separate analyses for active interventions. For the analyses of the follow-up effects, we used the comparison with the psychoeducation group, on the basis of which intervention was used more commonly for the treatment of depression/anxiety [30].

### Quality of included studies

CTAM scores ranged from 29 to 82 (Table 1, last column), with only three out of eighteen studies considered to be of adequate quality, with a CTAM score of at least 65 [79, 90, 93].

Of the eighteen RCTs, all but one [83] started out with true randomization (of which six did not describe the process of randomization [80, 81, 82, 85, 91, 94]). One study became quasi-randomized throughout the trial, as participants were moved to the control group after randomization because of inability to do yoga [84].

Six studies reported the use of independent assessors who were blind to group allocation [79, 80, 85, 87, 90, 93]. In all but three studies the intervention was adequately described [78, 93, 94], with ten of these using a treatment manual [79–82, 86, 87, 89–92]. In four studies, the dropout was not reported [78, 80, 81, 85]. In the other studies, the dropout ranged from 0% to 47%, with a mean dropout rate of 18%. At follow-up, dropout rates ranged from 14% to 67%, with a mean of 26%. One study had a dropout rate of over 50% (67%, at one-year follow-up [34]). Five studies used intention-to-treat analysis [89, 90, 92–94]. When intention-to-treat data were available, they were used for the assessment of effect sizes.

### Analysis of the overall effects

#### Depression: Yoga vs. an active control condition (all, psychoeducation, and other) or TAU

**Fig 2:**
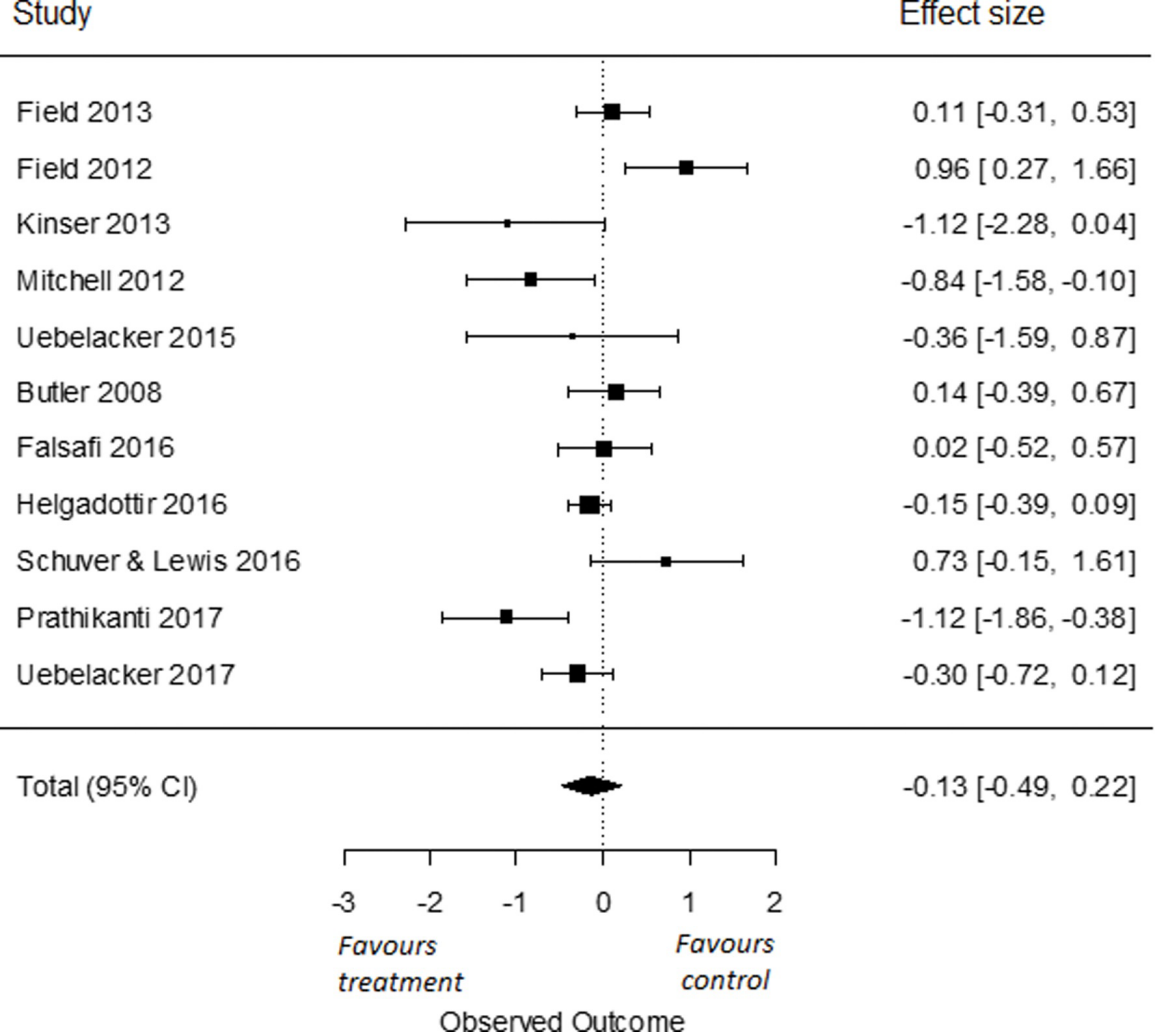
Effect of yoga versus all active control conditions on depressive symptoms.

To compare the effects of yoga on symptoms of depression compared to an active control group, we used all eleven RCTs from which we had data [35, 79–82, 86–90, 93]. We found an overall effect size of Cohen’s *d* -0.13 (95% *CI* = -0.49, 0.22). This effect was not significant (*p* = 0.47; Fig 2). I^2^ was high and estimated at 77% (95% CI = 43, 94). The q-statistic assessing heterogeneity was significant (*Q*(10) = 29.78, *p* < 0.001), indicating that there was a significant heterogeneity amongst the included studies.

**Fig 3:**
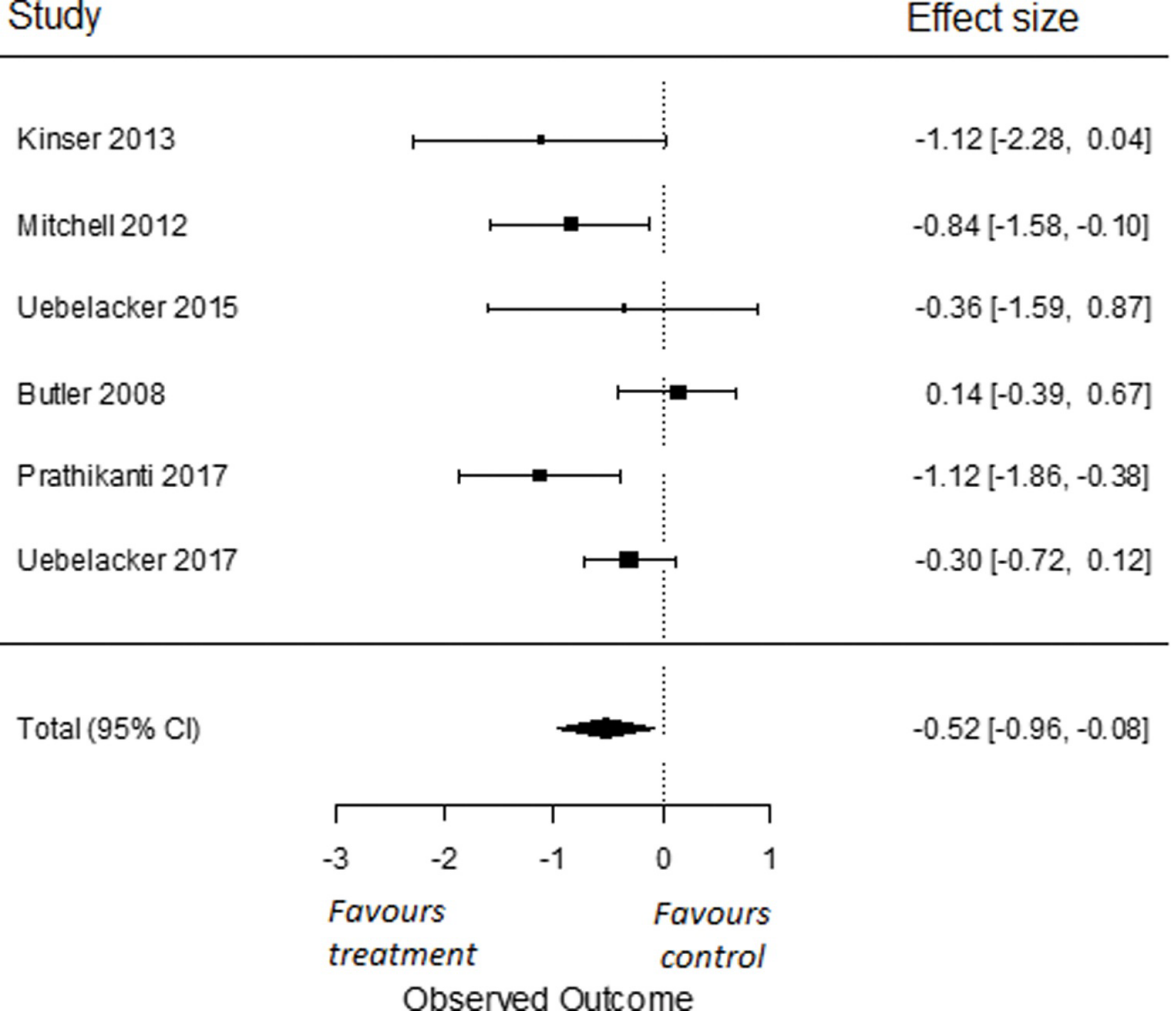
Effect of yoga versus psychoeducation control conditions on depressive symptoms.

**Fig 4:**
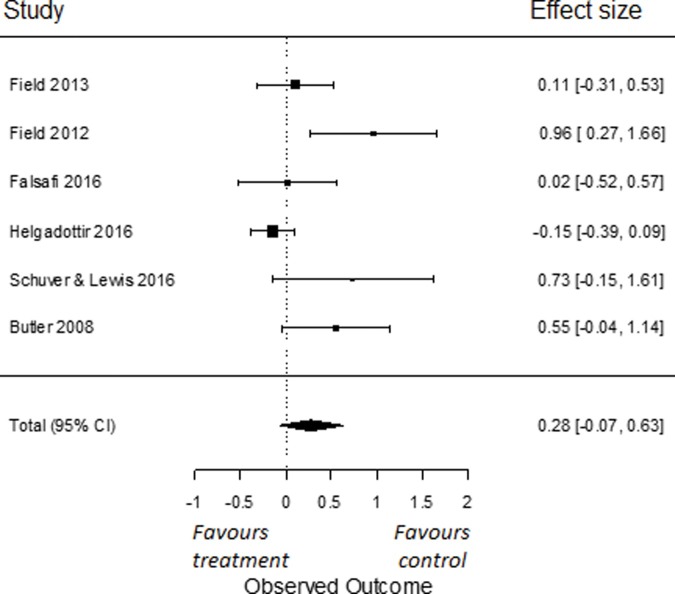
Effect of yoga versus other active control conditions on depressive symptoms.

In our sub-analyses, to compare the effects of yoga on symptoms of depression to a psychoeducation control group, we used all six RCTs for which data were obtained [81–82, 86, 87, 89, 90]. We found an overall effect size of Cohen’s *d* -0.52 (95% *CI* = -0.96, -0.08). This effect was significant (*p* = 0.02; Fig 3). I^2^ was moderate and estimated at 56% (95% CI = 0, 92). The q-statistic assessing heterogeneity was not significant (*Q*(5) = 10.71, *p* = 0.06), indicating that there was no significant heterogeneity amongst the included studies. To compare the effects of yoga on symptoms of depression compared to other active control groups, we used all six RCTs from which we had data [35, 79, 80, 87, 88, 97]. We found an overall effect size of Cohen’s *d* 0.28 (95% *CI* = -0.07, 0.63). This effect was not significant (*p* = 0.12; Fig 4). I^2^ was moderate to high and estimated at 65% (95% CI = 8, 95). The q-statistic assessing heterogeneity was significant (*Q*(5) = 14.31, *p* = 0.01). This indicates that the included studies were heterogeneous.

**Fig 5:**
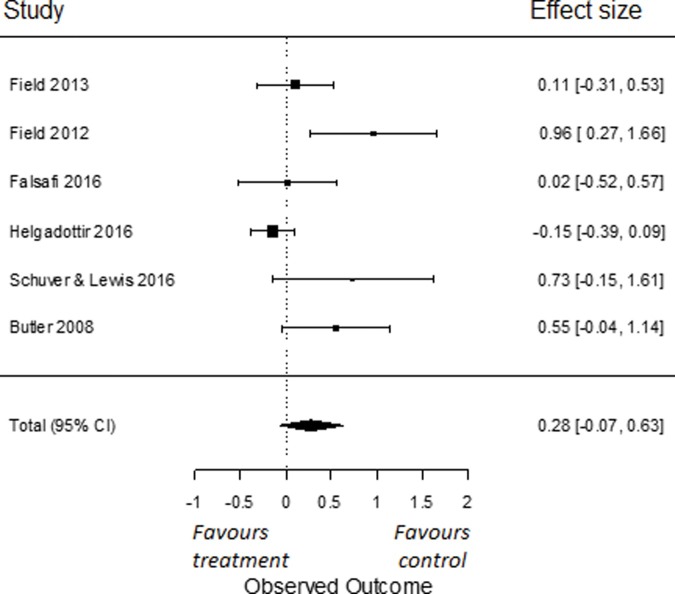
Effect of yoga versus TAU on depressive symptoms.

To compare the effects of yoga on symptoms of depression compared to TAU, we used five RCTs [80, 88, 92–94]. The overall effect size found was Cohen’s *d* -0.64 (95% CI = -1.41, 0.13), which was not significant (*p* = 0.10; Fig 5). I^2^ was high and estimated at 93% (95% CI = 76, 99). The q-statistic assessing heterogeneity was significant (*Q*(4) = 27.51, *p* < 0.001), again indicating significant heterogeneity amongst studies. As we found only two RCTs assessing symptoms of depression in chronic and/or treatment-resistant patients, no separate analyses for the chronic group were performed.

**Fig 6:**
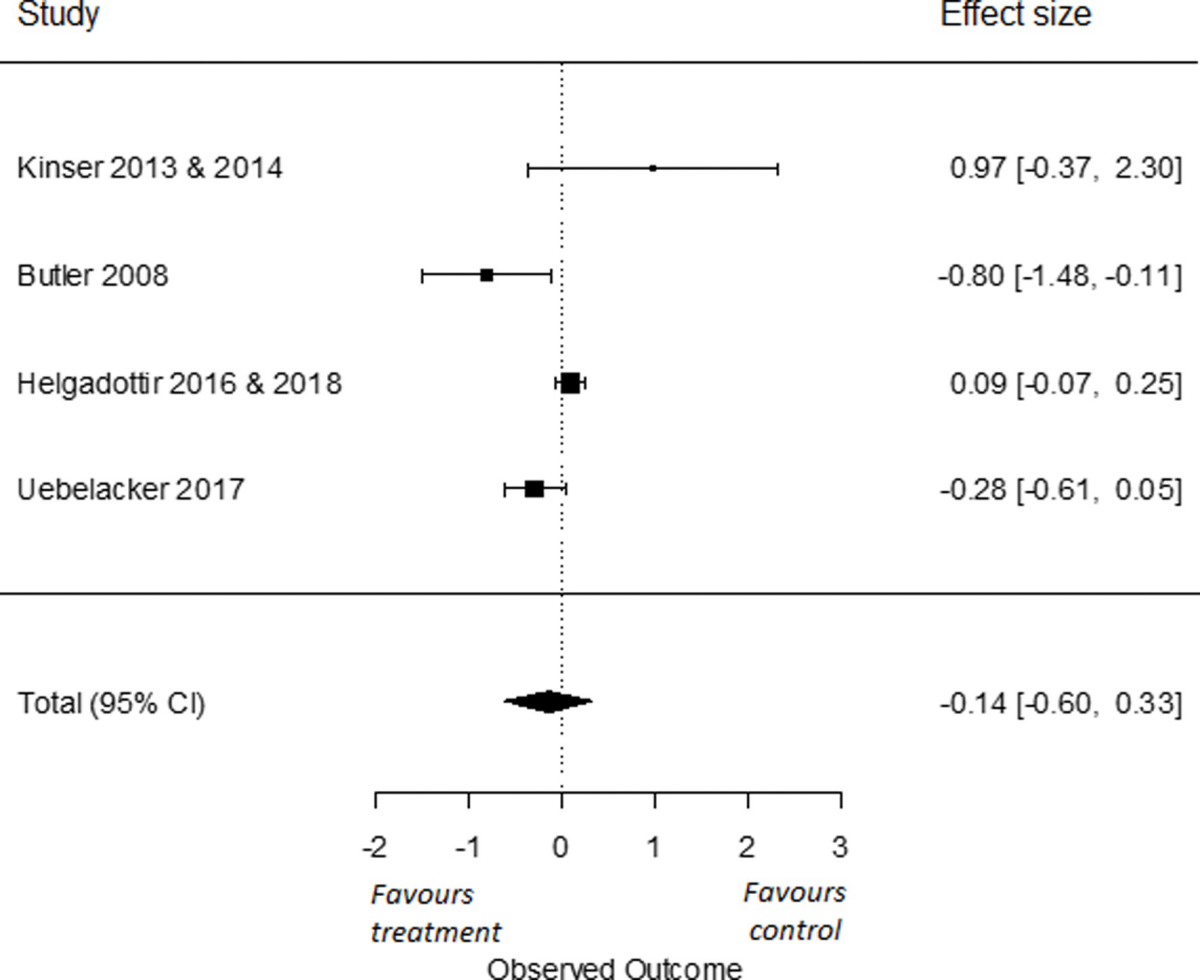
Long term effects of yoga versus active control conditions on depressive symptoms.

To compare the long-term effects of yoga on symptoms of depression to an active control group, we used four RCTs that had data from a follow-up of six months or longer [34, 87, 90, 97]. We found an overall effect size of Cohen’s *d* -0.14 (95% *CI* = -0.60, 0.33). This effect was not significant (*p* = 0.56; Fig 6). I^2^ was high and estimated at 78% (95% CI = 17, 99). The q-statistic assessing heterogeneity was significant (*Q*(3) = 11.35, *p* = 0.01), indicating that there was a significant heterogeneity amongst the included studies.

#### Anxiety: Yoga vs. an active control condition

**Fig 7:**
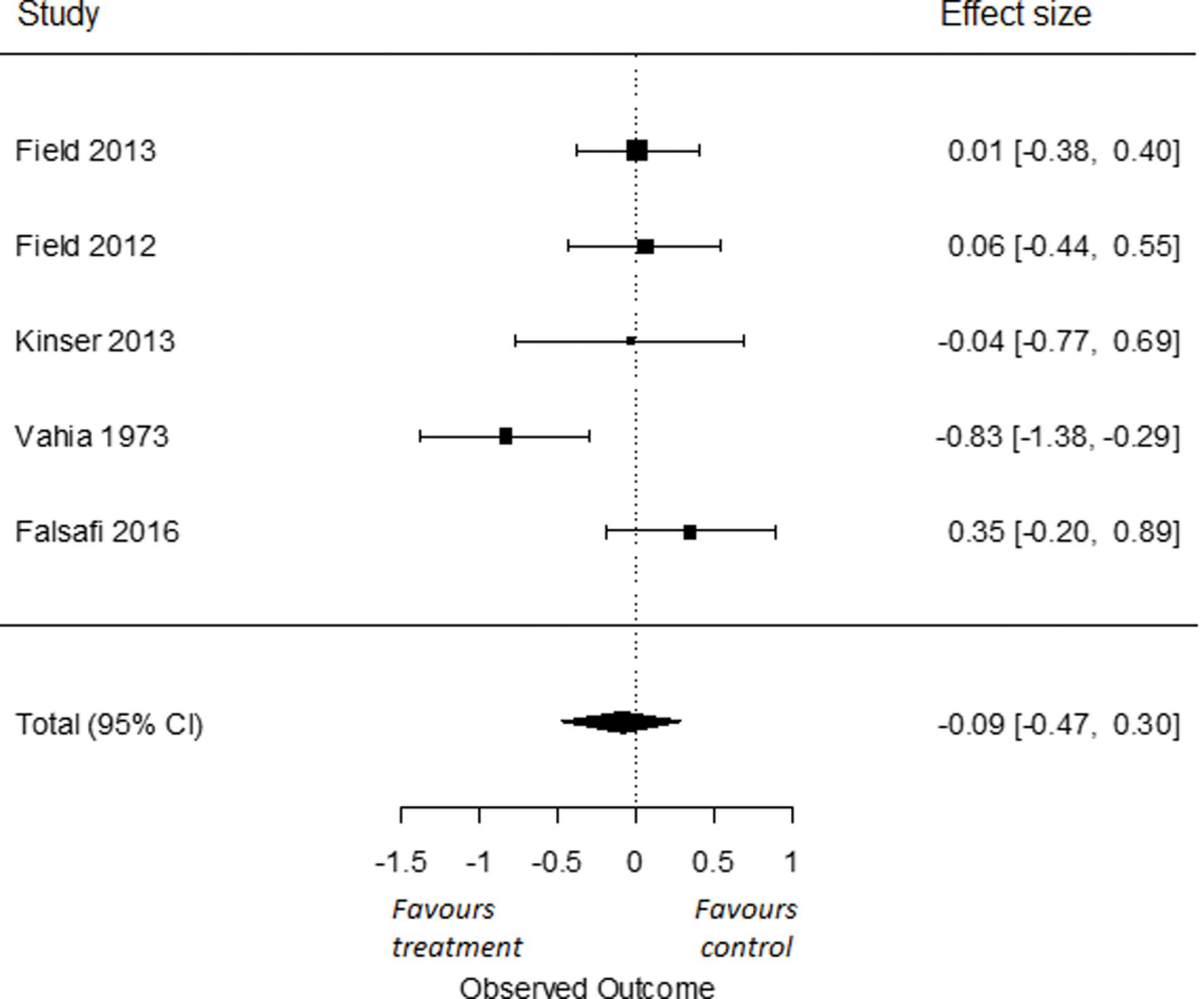
Effect of yoga versus active control condition on symptoms of anxiety.

To compare the effects of yoga on symptoms of anxiety compared to active control groups, we used all five RCTs for which data were obtained [79, 80, 85, 86, 88]. The overall effect size was Cohen’s *d* -0.09 (95% *CI* = -0.47, 0.30), which was not significant (*p* = 0.65; Fig 7). I^2^ was moderate to high and estimated at 63% (95% CI = 0, 96%). The q-statistic assessing heterogeneity was significant (*Q*(4) = 10.34, *p* = 0.04). This indicates that the included studies were heterogeneous. As we found only five RCTs for anxiety, no separate analyses for yoga versus TAU, psychoeducation or other active control groups were performed. Also, we found only one RCT assessing symptoms of anxiety in chronic and/or treatment-resistant patients and only one RCT assessing effects after a follow-up of six months or more; therefore, no separate analysis for these groups were performed.

### Publication bias

**Fig 8:**
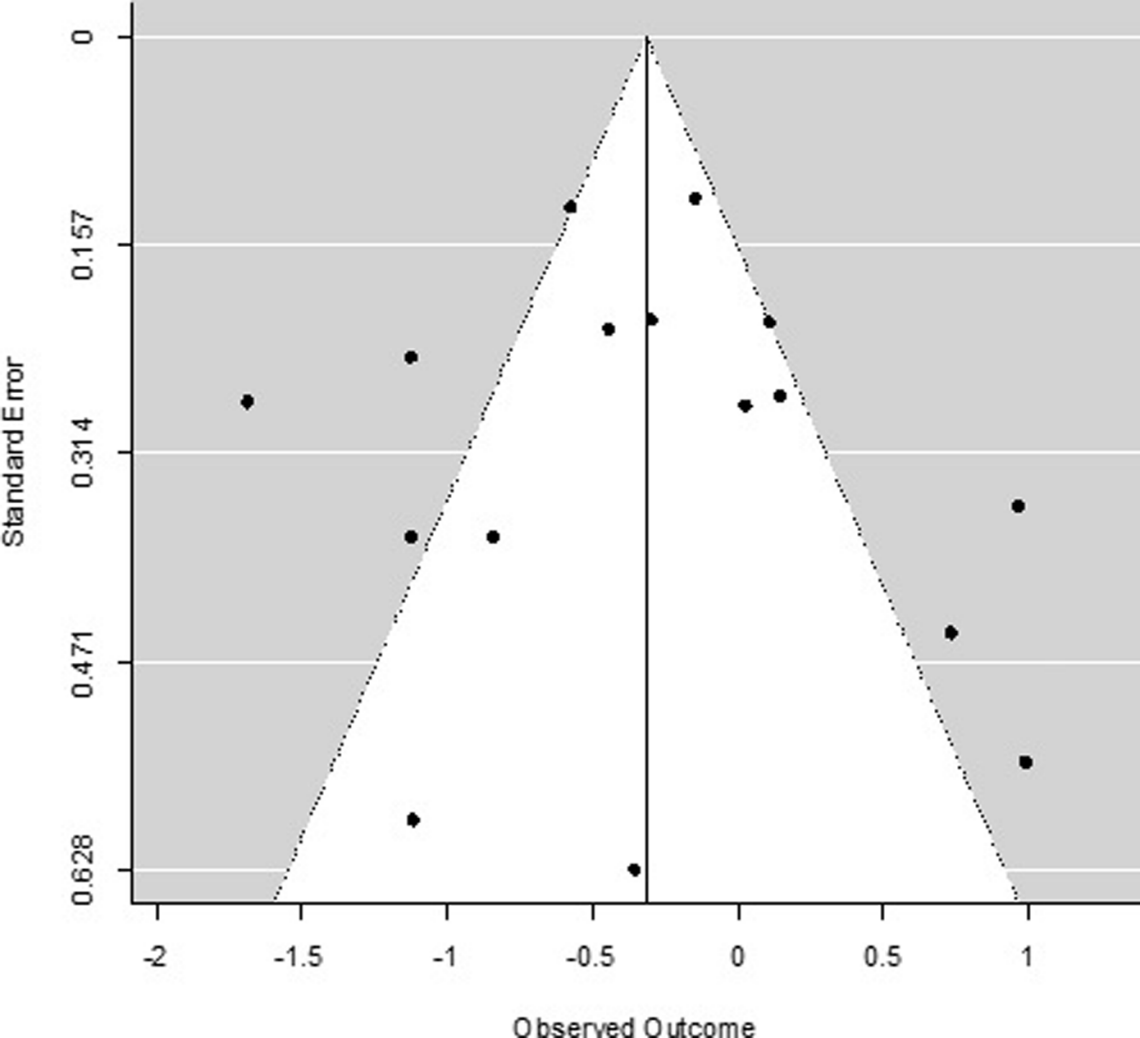
Funnel plot of included studies for depression.

**Fig 9:**
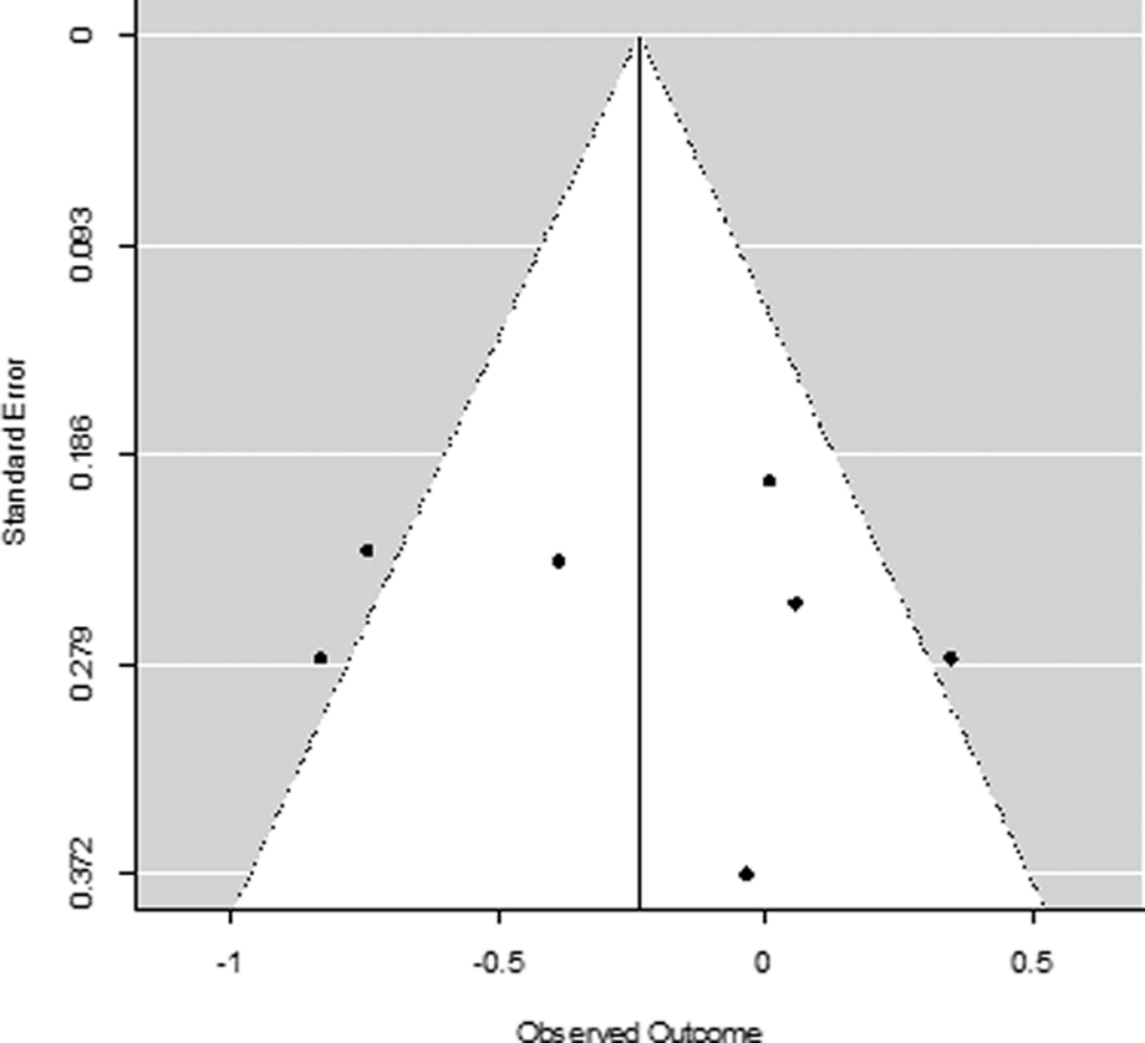
Funnel plot of included studies for anxiety.

Due to the low number of studies and the significant amount of heterogeneity, the Egger’s test for small-study effects was not computed in line with recommendations for meta-analyses [104]. Instead, publication bias was visually inspected using funnel plots. The funnel plot of studies examining the effect of yoga versus control conditions (both active and TAU) on depression (Fig 8) shows some evidence of funnel plot asymmetry, which may indicate a publication bias but could also be a result of significant heterogeneity amongst studies. Similarly, the funnel plot for studies examining the effect of yoga versus a control condition on anxiety (Fig 9) demonstrates some indication of funnel plot asymmetry, again due to either publication bias or heterogeneity amongst studies. Given the low number of studies in this meta-analysis, we cannot draw formal conclusions on the presence of a publication bias.

### Qualitative description

#### Effects for chronic and/or treatment-resistant populations

The number of studies in chronic and/or treatment-resistant patients was too low to perform separate analyses for the meta-analysis. As the possible effect of yoga for this population is important, we qualitatively reviewed the effects found in the four studies [83, 85, 87, 90]. One study was conducted with 52 patients with dysthymia, double depression and/or major depressive disorder, being symptomatic for over two years without significant remission of two months or more [87]. These authors found no significant difference between the yoga group and control group (psycho-education sessions) at six and nine months post-treatment (a 12-week intervention) on level of symptoms of depression. At nine months post-treatment they did find a significant difference in number of remissions between the yoga group and the control group with more patients in the yoga group reporting a remission (defined as not having a mood disorder of at least two months) with a medium-large effect size of -0.41 (Cramer’s *V)*. They also found that in the yoga group, compared to the control group, fewer people were in a new major depression episode. Although this difference was not significant at the level of *p* = 0.05, they found a medium effect size of 0.34 (Cramer’s *V*).

One study included 122 patients, with two thirds of the sample having chronic major depression, reporting symptoms over the past two years with absence of remission over two months [90]. The results indicated no significant difference between the yoga group and control group (health education classes) at post-treatment (ten weeks) on level of symptoms of depression. At six months follow-up there was a significant difference favoring the yoga group with a medium effect size (Cohen’s *d* of 0.50) on level of symptoms of depression. The study found no significant difference of full remissions between the yoga and control groups at six months follow-up.

One study included 27 patients with treatment-resistant psychoneurosis or depression, without response to conventional treatments [85], and found a significant difference in symptoms of anxiety between the yoga group and control group (relaxation resembling yoga) four weeks post-treatment in favor of the yoga group with a large effect size of 1.63 (Cohen’s *d*). One study included 12 patients of whom the majority were diagnosed with chronic generalized anxiety disorder (3–5 years of disorder) and found no difference in symptoms of anxiety between a yoga group and a control group of naturopathy (various massage techniques and breathing practices) after 21 days of treatment [83].

These four studies showed that participants in a yoga intervention reported more remissions than a control group at 9-months follow-up, but not fewer symptoms of depression [87], more decrease in symptoms of depression compared to a control group at 6-months follow-up, but not immediately after the intervention [90], more decrease in symptoms of anxiety four weeks after the intervention compared to a control group of relaxation resembling yoga [85], and no difference between a yoga group and a control group of naturopathy [83]. Although the studies reported mixed results, most of them showed that yoga might have some promise for patients with chronic forms of mood and anxiety disorder, especially at longer periods of follow-up.

## Discussion

This systematic review and meta-analysis was conducted to investigate whether hatha yoga is an effective treatment for acute, chronic and/or treatment-resistant mood and anxiety disorders. Through a systematic search we found eighteen studies investigating hatha yoga for mood and anxiety disorders, fifteen in acute patients and three in chronic and/or treatment-resistant patients. Three out of eighteen were of high methodological quality according to the CTAM [72]. The data of thirteen RCTs could be included in the analyses. Our findings showed no significant effect for hatha yoga on symptoms of depression compared to treatment as usual or compared to all active control groups. However, a comparison of yoga to psychoeducation control showed that hatha yoga led to reductions of symptoms of depression. For depression, at six months follow up or longer, we did not find a significant effect for hatha yoga compared to active control condition. Further, our findings show no significant effect for hatha yoga on symptoms of anxiety compared to active control groups.

Our results contrast with previous findings of yoga’s effectiveness in reducing symptoms in mood and anxiety disorders [51–59]. A previous meta-analysis on yoga for depression found medium-large effect sizes for yoga compared to usual care (standardized mean difference (SMD) of -0.69), for yoga compared to relaxation (SMD of -0.62), and for yoga compared to exercise (SMD of -0.59) [51]. An earlier meta-analysis on yoga for anxiety found small effect sizes for yoga compared to no treatment (SMD of -0.43) and large effect sizes for yoga compared to active control groups (SMD of -0.86) [59]. The discrepancy between these results and our own findings may be due to various methodological differences. First, the current meta-analysis focused on hatha yoga in contrast to both meta-analyses by Cramer et al. [51, 59], in which only six of the twelve studies [51] and seven out of eight studies [59] used hatha yoga. The Cramer meta-analyses are thus limited in their ability to draw conclusions specifically regarding hatha yoga interventions. It is possible that other forms of yoga are more effective for mood disorders. This is a conclusion drawn by Cramer et al. [51], who found that meditation-based yoga interventions were more effective than exercise-based yoga interventions. Second, the current meta-analysis focused on clinical samples whereas five of the twelve RCTs in Cramer et al. [51] and three out of the eight RCTs in Cramer et al. [59] used non-clinical samples, limiting the ability to generalize these findings to clinical populations. The discrepant findings between the current results and those of Cramer et al. could indicate that yoga is more effective in subthreshold symptomatology in the general populations and less effective in patients diagnosed with mood and anxiety disorders. Third, a number of studies in our meta-analysis either used samples including both mood and anxiety disorder patients [88, 93], or used a sample of homogenous participants (i.e., either mood or anxiety patients) but collected outcome measures for both mood and anxiety symptoms [34, 79, 80]. Given that participants in these samples were not required to have elevated levels of both depression and anxiety at baseline, they may have had lower scores on one measure, restricting the amount of room for improvement. However, we checked the mixed samples studies, and found that, on average, participants scored above the clinical cut-off scores on both anxiety and depression, leaving enough room for changes in the mean scores.

Considering the effects of hatha yoga for chronic and/or treatment-resistant populations, our qualitative review shows that participants in a yoga intervention reported more remissions than a control group at 9-months follow-up but no change in level of symptoms of depression at 9-months follow-up [87], more decrease in symptoms of depression compared to a control group at 6-months follow-up, but not immediately after the intervention [90], more decrease in symptoms of anxiety four weeks after the intervention compared to a control group [85], and no difference between a yoga group and a control group of naturopathy [83]. Given these mixed findings, conclusions are hard to draw but it seems that yoga interventions might be effective for patients with chronic and/or treatment resistant mood and anxiety disorders at a longer follow-up.

### Limitations of the current study

One important limitation on interpreting the results, consists of the quality of the studies included in the analyses. Specifically, only three of the eighteen studies had good methodological quality [79, 90, 93]. In the remaining fifteen studies, the quality was low to moderate due to a number of reasons such as a lack of (1) assessment by independent assessors, (2) assessment carried out blind to the treatment allocation, (3) intention-to-treat analysis, (4) an adequate description of the intervention or the use of a manualized intervention, and (5) insufficient sample size. Also, the results of three studies were not included in the meta-analysis as the authors did not provide the requested data. Further, the included studies were heterogeneous with regard to type of clinical population, amount and length of yoga practice, yoga ingredients, and control group. This heterogeneity was demonstrated in the I^2^ and Q-statistic of comparisons in this study, indicating the results should be carefully interpreted as each factor could moderate the meta-analytic effects. Given the low number of studies we were not able to investigate this potential moderation.

Moreover, several studies described the intervention as yoga without being specific regarding why the intervention was considered to be yoga-based. Although a prototypical hatha yoga practice would include instructions that elicit a mind state based on yoga theory (e.g., encouraging a mindful awareness, a connection between body and mind), it is possible that in some cases the yoga postures are administered essentially as stretching practices. We recommend that future publications include sufficient information about the yoga intervention so that potential instructional moderators can be examined. Another issue regarding interpretation is the heterogeneous types of control groups. Given that some of the control conditions have been found to be effective in treating depression (e.g., aerobic exercise and mindfulness), their inclusion as active controls will lessen the likelihood of finding effects for yoga. We found evidence for this perspective in our sub-analysis that omitted control groups that clearly involved a therapeutic element over and above non-specific factors (i.e., mindfulness, aerobic exercise, walking, and a group therapy intervention) in order to compare hatha yoga with psychoeducation. The results indicated that compared to psychoeducation controls, hatha yoga showed a benefit on depression symptoms.

One more factor of importance is that, only five of the included interventions used yoga programmes designed specifically for patients with mood and anxiety disorders [35, 82, 86, 89, 92]. This might limit the effectiveness of yoga interventions in general. For example, findings have shown that a mindfulness intervention designed specifically for depressed patients (MBCT) was effective in reducing symptoms of depression in patients with acute disorders where a general mindfulness intervention (MBSR) was not [105]. This suggests potential problems in using more general yoga interventions for treating mood and anxiety disorders, and emphasizes a need to study yoga interventions that are specifically designed to target mood and anxiety disorders.

A last limitation of note is that we had too few studies to examine publication bias using Egger’s tests. Instead, funnel plots were visually inspected, which indicated a slight asymmetry. Although this asymmetry could imply publication bias, this meta-analysis has too little studies to draw formal conclusions on this matter. This is a significant limitation as a presence of publication bias would imply our results cannot be reliably interpreted. Given the funnel plots indicated a slight publication bias, yet cannot be formally inspected due to the small *N* of this study, we again urge that our results are interpreted with caution. More high-quality research is needed to reliably determine the effect of yoga on depressive/anxiety symptoms compared to TAU and active control interventions.

### Clinical implications and further research

Our main conclusion is that there is not enough solid evidence for hatha yoga to be considered an effective treatment for mood and anxiety disorders. Therefore, no recommendations for clinical practice can be made. More RCTs are needed using methodology in accordance with the Clinical Trials Assessment Measure for psychological treatments [72] to examine the effectiveness of hatha yoga interventions for the treatment of acute, chronic and/or treatment-resistant mood and anxiety disorders. Such studies would benefit from making use of the guidelines of conducting clinical trials as described in the CONSORT statement [106]. We also recommend the publication of studies using open access and making the data online available.

## Supporting information

S1 File PRISMA 2009 checklist. (DOCX)
