# Correction: Hatha yoga for acute, chronic and/or treatment-resistant mood and anxiety disorders: A systematic review and meta-analysis

**DOI:** 10.1371/journal.pone.0216631

**Published:** 2019-05-02

**Authors:** The PLOS ONE Staff

Fig 5 is incorrectly printed as a duplicate of Fig 4. The publisher apologizes for the error. Please view Fig 5 here.

**Fig 5:**
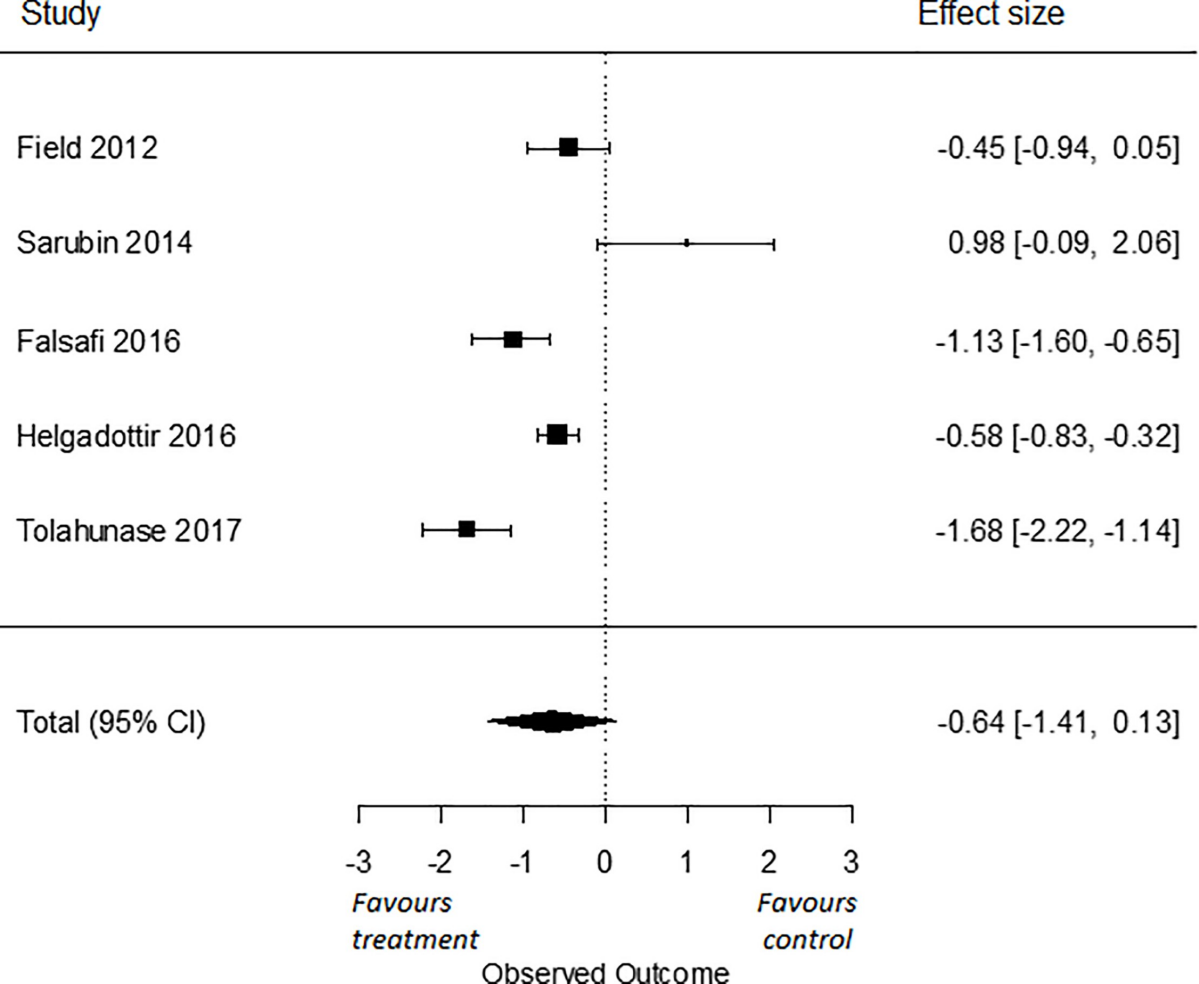
Effect of yoga versus TAU on depressive symptoms.
